# A Prosthodontic Approach as a Complementary Solution for a Complicated Orthodontic Treatment of a Patient with Cleidocranial Dysplasia

**DOI:** 10.1155/2021/6618813

**Published:** 2021-07-06

**Authors:** Joanna Jasnoch, Maria Zielke, Izabela Maciejewska

**Affiliations:** Department of Dental Prosthodontics, Medical University of Gdansk, Poland

## Abstract

This clinical report describes a prosthodontic rehabilitation of a 29-year-old patient with cleidocranial dysplasia (CCD), who, after completing an orthodontic treatment, was not satisfied with the aesthetic outcome. Besides aesthetics, the patient complained about mastication muscles pain, and clicking while eating but was not aware about her unilateral open bite on the right side. The aim of this treatment was to improve smile appearance and patient's well-being, as well as to restore the proper occlusal vertical dimension (OVD) along with complete intercuspation and to establish masticatory function. The first phase of the treatment concentrated on eliminating the muscle pain and temporomandibular joint (TMJ) clicking with a repositioning splint. During the second phase, the functional and aesthetic rehabilitation was obtained using adhesive prosthesis overlays and veneers.

## 1. Introduction

Cleidocranial dysplasia (OMIM #1190600; CCD) is a rare congenital autosomal disorder. Human genetics confirmed all kinds of mutations (dominant, recessive, and germ line mosaicism) in the RUNX2 sequence as the reason of the CCD [1–4]. Since RUNX2 (human 6p21) is a critical factor for both bone and cartilage development, the phenotype of CCD results from a disturbance in the intramembranous ossification, predominately, the skull and clavicles, but in severe cases, the entire skeleton might be affected [5, 6]. Individuals diagnosed with CCD are short and have either undeveloped or hypoplastic, thus hypermoving clavicles. The skull is brachycephalic with pronounced frontal and parietal bossing, midface hypoplasia, wide base of the nose, and depressed nasal bridge, coming in concert with the ocular hypertelorism. Severe dental abnormalities, which appear as a chaotic dentition, have been reported [7]. The most distinctive dental manifestations are the prolonged retention of deciduous teeth and delayed eruption of permanent teeth, which are impacted in the jaw bone next to the multiple supernumerary teeth [8]. The latter sporadically produce even a full set of additional dentition [7]. Due to the delayed eruption of permanent teeth and the limited space in the bone resulted from the existence of supernumerary teeth, the development of permanent teeth might be affected, reflecting ectopia and aberrant morphology especially in the roots, including taurodontism [9]. Thus, dental treatment of CCD positive patients is challenging and compels an interdisciplinary approach including surgery, orthodontics, conservative dentistry, and prosthodontics. It usually begins at youth, takes years, and beside an appropriate function, should address the patient's aesthetic expectations.

The aim of this paper was to present a prosthetic approach to the treatment of an adult patient with the CCD phenotype who suffered from the complicated outcome of the orthodontic treatment and was seeking the “profound improvement of her smile.”

## 2. Case Presentation

**Figure 1:**
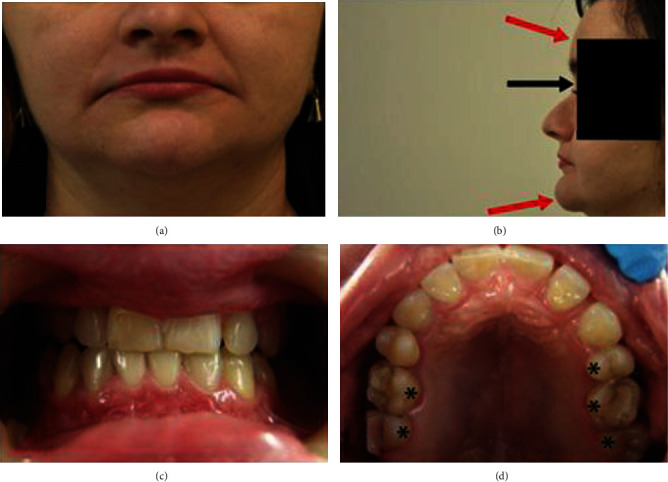
A 29-year-old patient with CCD. (a) Frontal view shows lowering corners of mouth and vermilion as well as deepening nasal-lip and chin wrinkles. (b) Profile view shows depressed nasal bridge (black arrow), frontal bossing, and prominent chin (red arrow). (c) Intraoral view before treatment shows disturbed proportions between height and width of maxillary anterior teeth with signs of tooth wear on incisal edges and posterior (right side) open bite. (d) Intraoral occlusal view of maxillary arch before treatment reveals gaps between teeth 21, 22, and 23. Tooth germs 15 and 25 are missing. Caries lesions in posterior teeth were noticed and had to be treated before placing overlays (asterisk).

A 29-year-old patient, clinically diagnosed with cleidocranial dysplasia, was searching for a “smile improvement.” The patient was aware of all the limitations regarding the possible therapy due to her medical/dental condition, but still was determined to undergo any possible reconstruction to “make her smile visible”. She presented short height and hypoplastic clavicles. Her head was slightly brachycephalic and exhibited frontal and parietal bossing. The nares were flared while the base of nose was depressed. Hypertelorism was also noted. The palate was gothic. At the time of appearance, the teeth were aligned but the crowns of the maxillary teeth were longer in width than height and completely invisible below the vermilion. Thus, the patient could have been mistaken as edentulous, even with the wide smile. The lower facial third was notably shorter than the middle and upper ones, respectively (Figures 1(a)–1(d)). Moreover, the patient complained about the pain and noise in the area of both temporomandibular joints (TMJ) while opening the mouth. TMJ reciprocal clicks were audible in both joints at the very beginning of mouth opening.

**Figure 2:**
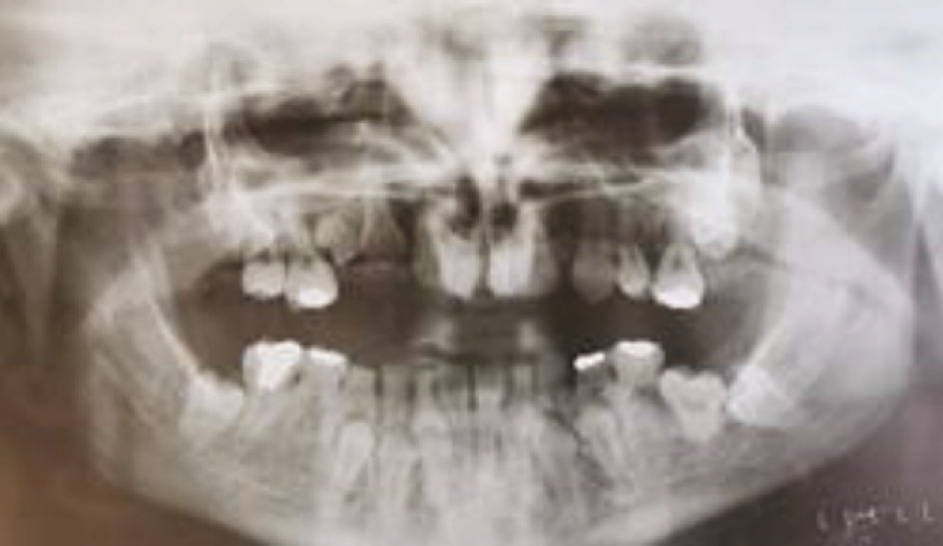
Panoramic radiograph taken at age of 16 before orthodontic treatment.

The patient's medical/dental history disclosed both orthodontic and surgical approaches, which began at the age of 16 due to the persistence of numerous deciduous teeth and unerupted permanent incisors, which significantly deteriorated her life quality. Panoramic radiographs revealed numerous imbedded permanent teeth in both arches and remaining deciduous teeth in the mandibular arch. In the maxilla, permanent teeth # 14, 13, 12, 11, 21, and 22 were imbedded, and both second premolars were lacking. In the mandible, there were persistent deciduous teeth # 75, 74, 73, 72, 82, 83, 84, and 85, imbedded permanent teeth #34, 33, 32, 42, 43, and 44, the lack of both second premolars, and all wisdom teeth. Despite the void of supernumerary teeth, the phenotype was specific for CCD (Figure 2) [8, 10]. According to the dental history, firstly, the embedded anterior permanent teeth were surgically exposed and orthodontically directed to the appropriate position in the maxilla. Fixed orthodontic appliance was used until the complete eruption, aligning, and leveling were achieved. Secondly, all primary lower teeth were extracted and the permanent teeth were exposed, followed by their alignment with the use of an orthodontic bracket. Although all permanent teeth were exposed in the mouth, lined, and set in the right position, the orthodontic treatment outcome did not meet the patient's aesthetic expectations.

**Figure 3:**
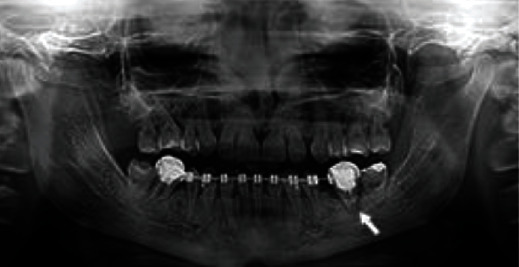
Panoramic radiograph taken at age of 29 right before prosthodontic treatment.

The dental diagnosis was set after a comprehensive clinical and radiological examination (Figure 3) as well as extraoral picture analysis, which showed smile disharmony in accordance with both horizontal and midface axes. Specifically, the incisal plane was not parallel to the interpupillary line but ascended about 2 mm on the right. During the natural smile, the upper teeth were not exposed at all; the upper lip descended slightly on the right. The dental and facial midlines did not corelate, and the mandibular midline was deviated to the right side. The radiograph revealed absence of maxillary and mandibular second premolars, all wisdom teeth, and many abnormalities in the length and shape of roots, including taurodontism. A periapical lesion around the tooth # 36 was noticed as well as widening of the periodontal fissure around tooth #46 with unsatisfying root filling. We assumed the problems with the mandibular first molar periodontium might have been caused by excessive orthodontic forces and the occlusal overload.

The patient requested a reconstruction of the upper dental arch, exclusively. Since the patient's critical expectation was to make her smile visible, the reconstruction required an elongation of the upper incisors up to 10 mm, which at that time was twice as long as they were. Thus, the final treatment plan was divided into two phases.

### 2.1. Phase 1: Temporary Repositioning Occlusal Splint

**Figure 4:**
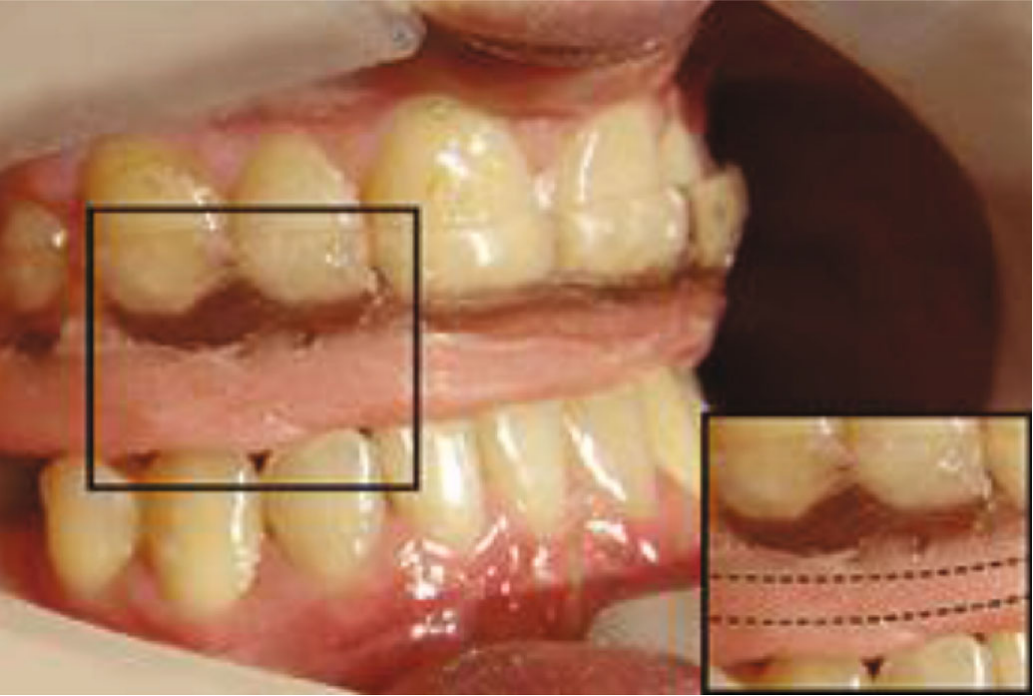
Occlusal repositioning splint used during first phase of prosthodontic treatment. In right corner, picture showing approximate thickness of each layer.

In the first phase, which took over 11 months, the patient used the repositioning occlusal splint, with the recommendation to use it during the night and also during the day as often as possible. The main purpose of this treatment was to deprogram the muscles and rebuild the new occlusion including an increased occlusal vertical dimension (OVD). For the bite registration, we used the Arcus Digma II system by KaVo. Since our main concern was not to overcome the patent's physiological tolerance, initially, the lateral height of the splint was 3 mm, which allowed to keep the OVD in the envelope of function. The patient accepted the new vertical dimension very fast and reported an improvement in the masticatory muscles' relaxation. After each 4 months, the splint height was enlarged sequentially by 2 mm, up to 7 mm final increase of the initial OVD at the lateral parts of the splint (Figure 4).

### 2.2. Phase 2: Mock-up and Definitive Restoration

**Figure 5:**
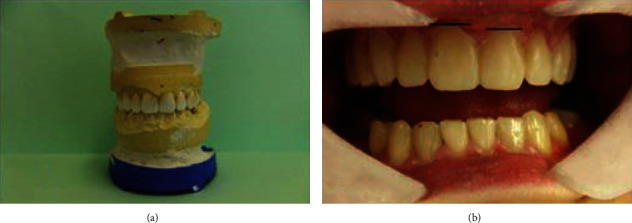
(a) Wax-up of maxillary arch. (b) Mock-up of maxillary arch, new occlusion was tested for over a month.

**Figure 6:**
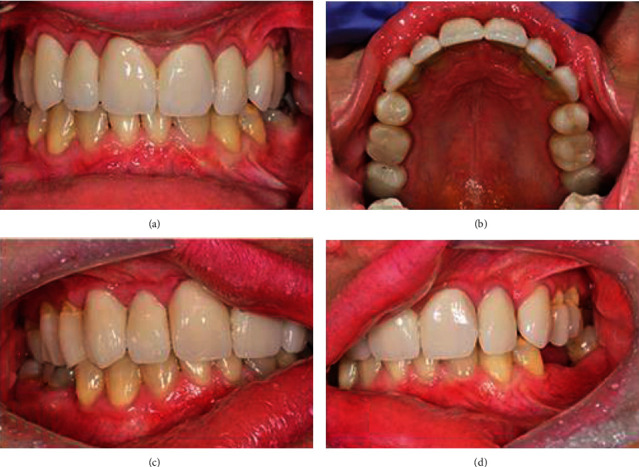
A 29-year-old patient with CCD. (a) Intraoral view of final restoration. It was patient's strong wish for restorations to be this bright, even though it did not match her natural teeth. (b) Intraoral occlusal view of maxillary arch after accomplished prosthodontic treatment. (c) Lateral view of the right side after 24 months follow-up. (d) Lateral view of the left side after 24 months follow-up.

After over 11 months of preliminary treatment, the patient reported a complete subside of the TMJ pain and full acceptance of the new OVD. Hence, the final treatment was initiated on the wax-up formation, and the mock-up was set on the upper dental arch (Figure 5). The patient did not consent to surgical crown lengthening to harmonise the gingival margins of central incisors (black bars in Figure 5 indicate disharmony). For over a month, the patient used the mock-up, which increased the OVD about 7 mm and elongated the anterior teeth up to about 10 mm without any complains. Thus, after the next 4 weeks, the occlusal situation was checked with the Arcus Digma II system, and the final restorations were prepared. The posterior teeth (17, 16, 14, 24, 26, and 27) were prepared for the composite overlays, while the anterior teeth (13, 12, 11, 21, 22, and 23) for the ceramic veneers (Figures 6(a)–6(d)). Since the elongation of the incisors' crowns might have jeopardized the crown-to-root ratio, to enhance the stabilization of the anterior teeth, the orthodontic retainer was placed along the palatal surfaces of the teeth: 13, 12, 11, 21, 22, and 23 (Figure 6(b)). The patient was provided with the relaxation splint of the minimal ≤2 mm thickness to prevent the lower teeth from potential abrasion until completion of the lower arch rehabilitation. Unfortunately, since the patient was almost completely satisfied with the treatment of the upper arch, she eventually refused the lower arch reconstruction, so was warned that lack of the lower arch rehabilitation might cause future complications such as wear of the lower teeth.

To our surprise, even though the anterior teeth were elongated up to 10 mm and protruded the upper lip by about 2 mm, the patient claimed she would ask for an additional treatment (hyaluronic acid injection) to lift the upper lip to make the anterior teeth even more visible.

**Figure 7:**
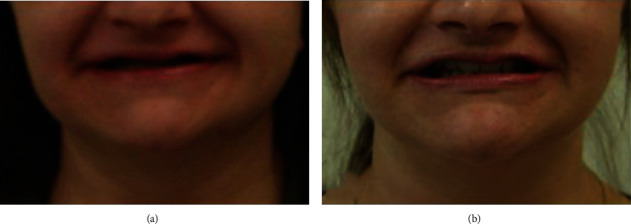
Comparison of facial appearance before (a) and after (b) treatment. (a) While smiling, maxillary teeth are not visible, giving impression that patient is edentulous. (b) Anterior teeth are visible during smile and dental midline correlates with facial midline.

**Figure 8:**
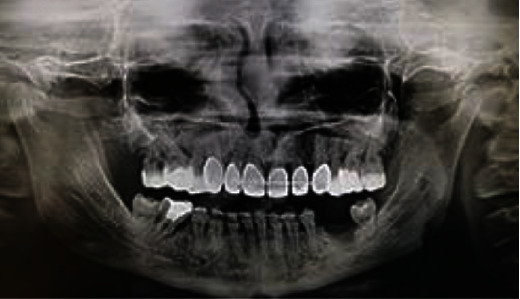
Panoramic radiograph on a follow-up appointment after 24 months.

After 24-month observation, the entire reconstruction acted steadily (Figures 7(a) and 7(b)). A panoramic radiograph was taken for evaluation of periodontal soft and hard tissues and search of the potential periapical lesions (Figure 8). The tooth #36 had to be extracted due to a failed endodontic treatment, which the patient decided to conduct at her residence area due to pandemic reasons. At the same clinic, the patients underwent the conservative intervention in the cervical region of teeth # 24 and 26, which unfortunately exceeds the natural contour of the teeth. The patient request was to match the color of her restoration and natural tooth (Figure 6(d)). On the follow-up appointment, the elongated teeth showed neither hypermobilty nor hypersensitivity, and teeth vitality was followed with the ethylium chloride. The proper occlusal contacts (central and lateral) were checked with the 40 *μ*m multicolor Bausch's articulation paper during every control appointment, and the ABC contacts were accepted as a reference point of the occlusion stability. From the patient's point of view, we followed any potential incidents of pain and/or muscle hypertension and TMJ clicking. During the observation time, none of the above was reported. The dental occlusion was stable and the patient did not report any muscle tension or TMJ clicking.

## 3. Discussion

Early diagnosis of the patients with CCD and subsequent surgical and orthodontic interventions lead to the best orthognathic and orthodontic results. Sometimes, although delayed therapy and/or disrupted teeth development jeopardize the final outcome, thus, additional prosthetic intervention becomes indispensable. CCD, being a rare disorder, causes many uncertainties as no treatment guidelines are set. Thus far, the described approaches are concentrated on the close cooperation between a dental surgeon and an orthodontist and the suggestion that the treatment should start around the age of 9 [11, 12]. It is conceivable that the beginning of the therapy at the age of 16 limited only to surgery and orthodontics burdened the entire procedure with cardinal failure, which cost the patient a long-term struggle with masticatory disfunction, TMJs pathology, and unsatisfactory aesthetics. After thorough prosthetic evaluation, it was discovered that patent's complains arose from significantly diminished OVD and the unilateral open bite on the right side. Thus, the treatment plan focused on achieving maximum aesthetics but with the appropriate reestablishment of the OVD.

It is commonly known that patients adapt relatively easy towards an increased OVD when it is risen in the in the physiological range [13, 14]. However, even in such cases, muscle adaptation requires an appropriate period of time. Weinberg [15], stated that masticatory muscles showed minimal activity while remaining in the rest position and concluded that increasing the OVD to this point might relieve a patient from pain. To meet our patient's needs, the OVD had to not only be elevated up to 7 mm but also the madibule had to be slightly shifted to the left, so following the gold standard, the repositioning occlusal splint was used as the first step of prosthetic rehabilitation [16]. Our main goal at that point was to precisely guide the condyles to a stable muscular-skeletal position, which allowed for harmonic muscle stretch and relaxation and gradually accustomed the patient to the new OVD. The atraumatic increase of the OVD during our CCD patient treatment might correlate with the alternative prosthodontic approach by Noh et al. [17], where the authors treated the CCD patient with the telescopic detachable prosthesis, which increased the OVD by 11 mm and resulted in the stable outcome. Another risk factor that had to be considered was the unfavorable rise of the crown-to-root ratio, which might have caused teeth mobility [18]. However, Lulic et al. [19] claimed that even the severe loss of periodontium when maintained by meticulous oral hygiene and rigid splinting resulted in longtime survival.

## 4. Conclusions

This clinical report presents a prosthetic rehabilitation of a patient with CCD, who was unsatisfied with her smile and suffered from masticatory muscle pain after a long and demanding surgical and orthodontic treatment. Ultimately, the prosthetic treatment established the new occlusal plane and increased the OVD, which resulted in visibility of the anterior teeth while smiling and at rest. Moreover, bilateral stabilization of the posterior occlusion with the composite overlays relieved the pain, thus restoring the proper function and creating a pleasing facial appearance according to the patient's expectations.
